# Psychological effects of being a healthcare worker and a caregiver for cancer patients: a comparative analysis

**DOI:** 10.1007/s00520-025-09699-w

**Published:** 2025-06-27

**Authors:** Aybeniz Civan Kahve, Dünya Gözde Çapar, Mustafa Kaan Keleş, Mehmet Alper Çınar, Gökhan Uçar, Perihan Perkin, Erol Göka

**Affiliations:** 1 Department of Psychiatry, Faculty of Medicine, Gazi University, Ankara, Turkey; 2 Department of Psychiatry, Ankara Bilkent City Hospital, University of Health Sciences, Ankara, Turkey; 3 Department of Medical Oncology, Ankara Bilkent City Hospital, University of Health Sciences, Ankara, Turkey

**Keywords:** Burnout, Professional, Caregivers, Health personnel, Neoplasms, Resilience, Psychological

## Abstract

**Purpose:**

Cancer caregiving presents significant psychological and emotional challenges. While healthcare workers (HCWs) possess medical expertise, their dual role as both professionals and caregivers may impose additional burdens. This study examines the psychological distress and caregiving burden among HCWs and non-HCWs caring for oncology patients.

**Methods:**

A cross-sectional study was conducted with 83 caregivers (46 HCWs, 37 non-HCWs) of oncology patients receiving treatment at a tertiary hospital. Participants completed sociodemographic surveys and psychological assessments, including the Hamilton Depression and Anxiety Scales, Maslach Burnout Inventory, and Resilience Scale for Adults.

**Results:**

Both HCWs and non-HCWs exhibited significantly higher depression and anxiety levels compared to healthy controls. No significant differences were found in burnout or psychological resilience between caregiver groups. However, HCWs reported additional stress due to professional expectations, managing medical decisions, and coordinating care. Despite their expertise, HCWs expressed a strong need for emotional and informational support.

**Conclusion:**

HCW caregivers face unique role conflicts and burdens beyond those of non-HCWs. Addressing their specific challenges through tailored psychosocial interventions and institutional support is crucial for improving caregiver well-being and patient care. Future research should explore long-term coping mechanisms and support strategies for HCW caregivers in oncology settings.

## Introduction

Cancer is one of the leading causes of death worldwide, responsible for approximately one in six deaths annually. According to data from the World Health Organization, nearly 10 million people died from cancer in 2020, with lung cancer identified as the most common cause of cancer-related mortality. Beyond its high mortality rate, cancer has profound physiological, psychological, and social impacts, not only on patients but also on those who care for them throughout the often complex and prolonged treatment journey [1].

With advancements in medical technology and treatment modalities, survival rates for many types of cancer have improved significantly. However, these improvements have also brought about new challenges, particularly concerning the prolonged caregiving needs of patients undergoing long-term oncological treatments [2]. Caregivers—who are often close family members or friends—are indispensable in ensuring that patients’ physical, emotional, and social needs are met during the treatment process [3, 4]. The concept of a caregiver is typically defined as an individual who provides ongoing physical and emotional support without financial compensation, assuming responsibilities that range from managing daily self-care tasks to offering psychological comfort [5].

A growing body of literature has highlighted the immense burden placed on caregivers of individuals with cancer. The physical demands of caregiving, combined with emotional stressors, often lead to symptoms such as fatigue, sleep disturbances, difficulty concentrating, and emotional exhaustion [6, 7]. Moreover, studies emphasize the reciprocal relationship between the health of the patient and the caregiver: as the psychological well-being of one declines, the other is likely to suffer as well, creating a cycle of shared distress [8]. In this context, caregiver burden is not only a matter of individual well-being but also directly influences the overall treatment process and outcomes for cancer patients. An especially underexplored group within this dynamic are caregivers who are themselves healthcare workers (HCWs). Boumans and Dorant conducted a study highlighting that HCWs who also provide informal care to family members—termed “double-duty caregivers”—experience significant mental and physical health challenges [9]. Their research demonstrated that as these professionals dedicate more hours to informal caregiving, they face increased emotional exhaustion, greater need for recovery, and more pronounced work-home and home-work interferences. Despite their professional expertise, these individuals often encounter blurred boundaries between their work and personal lives, leading to heightened stress levels [10, 11]. This underscores the necessity for further research into the unique psychological impacts faced by HCWs serving as informal caregivers.

In light of these considerations, the present study aims to evaluate the psychological burden experienced by HCW family members caring for oncology patients. Specifically, we aim to assess and compare levels of anxiety, depression, burnout, and psychological resilience between HCW caregivers and non-HCW caregivers. Additionally, this study seeks to identify the distinct challenges faced by HCWs in caregiving roles, explore the ways in which their professional background influences their caregiving experience, and contribute to the development of targeted support strategies. By understanding the multidimensional difficulties encountered by these caregivers, we hope to inform clinical practice and support the development of early interventions that promote mental well-being and reduce psychological distress. Ultimately, this research aims to shed light on an overlooked population: HCWs who, while dedicating their careers to caring for others, may face significant psychological strain when caring for their own loved ones.

## Material and methods

This cross-sectional study was conducted at the Adult Outpatient Chemotherapy Unit of Ankara City Hospital’s Oncology Department. Following ethical approval, over a period of approximately 6 months, patients receiving treatment were interviewed to identify their informal caregivers, with particular attention to whether any of them were HCWs (HCWs). In cases where both a HCW and a non-HCW relative were involved in caregiving for the same patient, this was recorded, and both individuals were invited to participate in the study. If the caregiver was present at the unit during the interview with the patient, they were informed about the study and invited to participate. Additionally, informative brochures describing the study and including contact information for the research team were distributed within the unit to reach potential participants. Those who contacted the researchers were subsequently invited to the hospital for face-to-face interviews. A total of 64 patients had a relative who was a healthcare professional. Among these, 18 patient-relative pairs were not included in the study due to unwillingness to participate. A total of 83 informal caregivers—46 HCWs and 37 non-HCWs, providing care to 46 cancer patients participated after providing informed consent. All participants completed the Resilience Scale for Adults, Hamilton Depression Rating Scale, Hamilton Anxiety Rating Scale, and Maslach Burnout Inventory. Healthy controls were selected from among hospital staff who volunteered to participate in the study, had no history or diagnosis of psychiatric disorders, and were not using any psychotropic medication.

### Measurement tools

Sociodemographic data form.

It was developed by the researchers to collect information on participants’ age, gender, education level, employment status, income, cohabitation with the patient, frequency of contact with the patient, and type of support provided. It also included questions on tobacco, alcohol, and substance use; chronic diseases and treatments; previous psychiatric history and medications; psychological support related to the oncology process; and history of suicide attempts. Additionally, data were collected on the cancer patient’s age, gender, cancer type, disease duration, stage, and current and previous treatments.

DSM-5 structured clinical interview form (SCID-5).

It was developed by First and colleagues; this interview was used to assess the presence of psychiatric disorders in participants. The validity and reliability study for use in our country was conducted by Elbir and colleagues in 2019 [12]. SCID-5 is a semi-structured diagnostic interview used to determine DSM-5 psychiatric diagnoses. It consists of several modules that assess different diagnostic categories such as mood disorders, anxiety disorders, psychotic disorders, and substance use disorders. The number of questions varies depending on the modules applied and the participant’s responses, but the full version contains more than 100 structured and branching questions. Each module begins with screening questions that determine whether more detailed follow-up questions are required. In our study, the SCID-5-CV (Clinician Version) was administered by a trained psychiatrist to assess the presence of current and past psychiatric disorders in participants.

Hamilton depression rating scale (HAM-D).

Developed by Hamilton in 1960, this scale was designed to measure the severity of depression. The validity and reliability study of the Turkish form was conducted by Akdemir and colleagues in 1996 [13].

Hamilton anxiety rating scale (HAM-A).

This scale, developed by Hamilton, assesses the severity of anxiety symptoms, including both psychic and somatic components. It also evaluates accompanying depressive symptoms. The Turkish validity and reliability study was conducted by Yazıcı et al. in 1998 [14].

Maslach burnout inventory (MBI).

Burnout, as defined by Maslach, is a syndrome of physical, emotional, and mental exhaustion, characterized by feelings of helplessness, hopelessness, and negative attitudes toward work and life. It consists of three dimensions: emotional exhaustion, depersonalization, and reduced personal accomplishment. The Maslach Burnout Inventory, developed by Maslach and Jackson in 1981, is a 22-item, 7-point Likert scale with three subscales. Its Turkish validity and reliability were established by Ergin and colleagues in 1992 [15].

The resilience scale for adults (RSA).

The scale consists of 33 items designed to assess various aspects of psychological resilience, organized into six subscales: self-perception, future planning, structured style, social competence, family cohesion, and social resources. Unlike many scales, it does not have a fixed minimum or maximum total score, nor does it include a cutoff point. Positive and negative statements are intermixed randomly on both sides of the scale to minimize response bias. Participants indicate their agreement by marking one of five boxes positioned between these statements. The scoring approach is flexible, allowing for measurement of resilience levels without rigid thresholds. This scale was originally developed by Friborg and colleagues, and its validity and reliability for the Turkish population were established by Basım and Çetin [16].

Considering the paper-based administration of the structured clinical interview (SCID-5) along with five psychometric scales, the average duration of the face-to-face interviews conducted with each participant was approximately 75 to 90 min.

### Statistical analysis

All statistical analyses were performed using the SPSS for Windows version 22.0 software package. Descriptive statistics were presented as means ± standard deviations for continuous variables and as frequencies and percentages for categorical variables. The normality of continuous variables was assessed using the Shapiro–Wilk test. For comparisons of categorical variables, the chi-square test was used; Fisher’s exact test was applied when chi-square test assumptions were not met, with Monte Carlo simulations employed for variables with small expected frequencies.

For continuous variables with normal distribution, independent samples *t*-tests were used to compare HCWs and non-HCWs. When comparing anxiety, depression, burnout, and resilience scores among HCWs, non-HCWs, and healthy controls, one-way ANOVA or Welch’s ANOVA was used depending on variance homogeneity, as assessed by Levene’s test. Post hoc pairwise comparisons were conducted using Tamhane’s T2 test when variances were unequal, and Tukey’s test otherwise. For variables violating normality assumptions or with ordinal data, non-parametric tests such as the Kruskal–Wallis test were used. A *p*-value of less than 0.05 was considered statistically significant.

## Results

Among the 83 participants included in the study, 53 (63.86%) were female and 30 (36.14%) were male, with a mean age of 42.05 ± 12.82 years. When age was compared between groups, HCWs were significantly younger (39.24 ± 10.05 years) than non-HCWs (45.54 ± 15.01 years; *t* = –2.189, *p* = 0.032). Educational attainment was also higher in the HCW group, with an average of 18.04 ± 3.55 years, versus 12.70 ± 4.75 years among non-HCWs (*t* = 5.684, *p* < 0.001), indicating a statistically significant difference. Detailed comparisons of sociodemographic characteristics and medical history between these groups are presented in Table 1. The healthy control group (*n* = 31) had a mean age of 32.03 ± 9.76 years, with females comprising 54.84% and males 45.16% of the group. Their mean education level was 14.74 ± 3.41 years. In terms of marital status, 45.16% were single and 54.84% were married.

**Table 1:** Comparison of healthcare workers and non-healthcare workers in terms of sociodemographic variables and medical history

		Healthcare workers (*n*:46)	Non-healthcare workers (*n*:37)	Total (*n*:83)	Statistical analysis
Gender	Female	32 (69.57%)	21 (56.76%)	53 (63.86%)	*Χ*^*2*^*: 1.458* *p: 0.227*
	Male	14 (30.43%)	16 (43.24%)	30 (36.14%)	
Age (mean ± SD)		39.24 ± 10.05	45.54 ± 15.01	42.05 ± 12.82	***T: − 2.189*** ***p: 0.032***
Education years (mean ± SD)		18.04 ± 3.55	12.70 ± 4.75	15.66 ± 4.89	***T: 5.684*** ***p* < *0.001***
Marital status	Single	14 (30.43%)	6 (16.22%)	20 (24.10%)	*Χ*^*2*^*: 2.267* *p: 0.132*
	Married	32 (69.57%)	31 (83.78%)	63 (75.90%)	
Child status	Have	32 (69.57%)	26 (70.27%)	58 (69.88%)	*Χ*^*2*^*: 0.005* *p: 0.945*
	Not have	14 (30.43%)	11 (29.73%)	25 (30.12%)	
Employment status	Working	41(89.13%)	20 (54.05%)	61 (73.49%)	***Χ***^***2***^***: 16.042*** ***p* < *0.001***
	Not working	0 (0%)	8 (21.62%)	8 (9.64%)	
	Retired	3 (6.52%)	7 (18.92%)	10 (12.05%)	
	Student	2 (4.35%)	2 (5.41%)	4 (4.82%)	
Income status	Very low	1 (2.17%)	2 (5.41%)	3 (3.61%)	***Χ***^***2***^***: 15.271*** ***p: 0.001***
	Low	0 (0%)	6 (16.22%)	6 (7.23%)	
	Moderate	9 (19.57%)	14 (37.84%)	23 (27.71%)	
	High	36 (78.26%)	15 (40.54%)	51 (61.45%)	
Living place	Family	44(95.65%)	35(94.59%)	79 (95.18%)	*p: 1.000*
	Single	2 (4.35%)	2(5.41%)	4 (4.82%)	
Living with cancer patient	Yes	11 (23.91%)	19 (51.35%)	30 (36.14%)	***Χ***^***2***^***: 6.689*** ***p: 0.010***
	No	35 (76.09%)	18 (48.65%)	53 (63.86%)	
Chronic illness	Yes	21 (45.65%)	19(51.35%)	40 (48.19%)	*Χ*^*2*^*: 0.267* *p: 0.606*
	No	25 (54.35%)	18(48.65%)	43 (51.81%)	
Smoking	Yes	16 (34.78%)	8 (21.62%)	24 (28.92%)	*Χ*^*2*^*: 1.728* *p: 0.189*
	No	30 (65.22%)	29 (78.38%)	59 (71.08%)	
Psychiatry disorder history	Yes	11 (23.91%)	5 (13.51%)	16 (19.28%)	*Χ*^*2*^*: 1.425* *p: 0.233*
	No	35 (76.09%)	32 (86.49%)	67 (80.72%)	
Suicidal attempt history	Yes	1 (2.17%)	1 (2.70%)	2 (2.41%)	*p: 1.000*
	No	45 (97.83%)	36 (97.30%)	81 (97.59%)	
Psychiatry disorder history in family	Yes	18 (39.13%)	9 (24.32%)	27 (32.53%)	*Χ*^*2*^*: 2.048* *p: 0.152*
	No	28 (60.87%)	28 (75.68%)	56 (67.47%)	

*n*, number of persons; *%*, percent; chi-square test was used for categorical variables. Fisher’s exact test was applied where appropriate. Monte Carlo simulation (100,000 samples) was used for variables that did not meet chi-square test assumptions

### Cancer patients

Among patients diagnosed with cancer and followed up in oncology, different cancer types and staging distributions were observed. The mean age of the cancer patients was 61.96 ± 13.31 years (min = 25, max = 87). Regarding cancer type, the most common malignancies were gastrointestinal system cancers (39.1%, *n* = 18), followed by genitourinary system cancers (19.6%, *n* = 9) and endocrine system cancers (17.4%, *n* = 8). Hematological malignancies accounted for 10.9% (*n* = 5), while respiratory system cancers were reported at 6.5% (*n* = 3). Central nervous system cancers constituted 4.3% (*n* = 2), and dermatological malignancies were the least common (2.2%, *n* = 1). Cancer staging analysis showed that the majority of patients were in advanced stages. Stage IV cancers were the most prevalent, accounting for 54.3% (*n* = 25) of cases. Stage III cancers comprised 30.4% (*n* = 14), while early-stage cases were less frequent, with stage II at 8.7% (*n* = 4) and stage I at 6.5% (*n* = 3).

### Depression scale scores

HAM-D scores indicated significantly higher depression levels in healthcare (*M* = 10.54, *SD* = 6.79) and non-HCWs (*M* = 9.35, *SD* = 6.60) compared to healthy controls (*M* = 2.45, *SD* = 2.66), with a significant group difference (*F* = 36.952, *p* < 0.001).

### Anxiety scale scores

HAM-A scores were highest among non-HCWs (*M* = 11.51, *SD* = 9.55) and HCWs (*M* = 10.89, *SD* = 6.93), with significantly lower anxiety levels in healthy controls (*M* = 3.55, *SD* = 2.72; *F* = 28.935, *p* < 0.001). Post hoc comparisons among the three groups revealed a significant difference, which was found to originate from the healthy control group (Table 2).

**Table 2:** Comparison of healthcare workers, non-healthcare workers, and healthy controls in terms of anxiety and depression scale scores

Scale	Healthcare workers	Non-healthcare workers	Healthy controls	Statistical analysis
*Hamilton Depression Rating Scale*	10.54 ± 6.79	9.35 ± 6.60	2.45 ± 2.66	*F: 36.952 p* < 0.001*
*Hamilton Anxiety Rating Scale*	10.89 ± 6.93	11.51 ± 9.55	3.55 ± 2.72	*F: 28.935 p* < 0.001*
*p-values were calculated using Welch’s ANOVA due to unequal variances across groups				
Variable	Compared groups		Mean difference	*p*-value**
*Hamilton Depression Rating Scale*	Healthcare workers	Non-healthcare workers	1.192	0.807
		Healthy controls	8.092*	** < 0.001**
	Non-healthcare workers	Healthy controls	6.900*	** < 0.001**
*Hamilton Anxiety Rating Scale*	Healthcare workers	Non-healthcare workers	− 0.622	0.983
		Healthy controls	7.343*	** < 0.001**
	Non-healthcare workers	Healthy controls	7.965*	** < 0.001**
**Pairwise comparisons were conducted using Tamhane’s T2 post hoc* test due to unequal variances*				

### Burnout levels

When comparing MBI scores among the groups, no statistically significant differences were found in emotional exhaustion (*F* = 0.743, *p* = 0.478), depersonalization (*F* = 1.742, *p* = 0.180), or total burnout scores (*F* = 0.185, *p* = 0.831). However, the personal accomplishment subscale demonstrated a borderline difference (*H* = 5.796, *p* = 0.055), with non-HCWs (11.59 ± 5.30) reporting higher personal accomplishment scores compared to HCWs (9.11 ± 4.18) and healthy controls (9.32 ± 4.06). Detailed comparisons of MBI scores are presented in Table 3.

**Table 3:** Comparison of Maslach Burnout Inventory scores among healthcare workers, non-healthcare workers, and healthy controls

Maslach burnout inventory	Healthcare workers	Non-healthcare workers	Healthy controls	Statistical analysis
Emotional exhaustion	13.70 ± 6.62	12.05 ± 7.32	12.06 ± 7.28	*F: 0.743 p*^*1*^*: 0.478*
Depersonalization	4.24 ± 3.03	3.14 ± 2.88	4.19 ± 2.75	*F: 1.742 p*^*1*^*: 0.180*
Personal accomplishment	9.11 ± 4.18	11.59 ± 5.30	9.32 ± 4.06	*H: 5.796 p*^*2*^*: 0.055*
Total score	27.04 ± 10.56	26.78 ± 10.51	25.58 ± 11.13	*F: 0.185 p*^*1*^*: 0.831*

*p*^*1*^, one-way ANOVA; *p*^*2*^, Kruskal–Wallis’s test

### Psychological resilience

When comparing the groups in terms of resilience, no significant differences were found in the total RSA score (*H* = 1.227, *p* = 0.542). Among the subscales, perception of self showed the highest score in healthy controls, but this difference was not statistically significant (*H* = 3.429, *p* = 0.180). Similarly, no significant differences were observed in structured style (*F* = 0.402, *p* = 0.670) or social competence (*F* = 1.045, *p* = 0.355) across the groups. Comparison of Resilience Scale for Adults (RSA) and subscales among HCWs, non-HCWs, and healthy controls is given in Table 4.

**Table 4:** Comparison of Resilience Scale for Adults (RSA) and subscales among healthcare workers, non-healthcare workers, and healthy controls

Scale	Healthcare workers	Non-healthcare workers	Healthy controls	Statistical analysis
RSA-Structured Style	11.13 ± 2.79	10.57 ± 3.19	10.71 ± 2.99	*F: 0.402 p*^*1*^*: 0.670*
RSA-Perception of the Future	11.11 ± 3.09	10.22 ± 4.14	11.32 ± 3.67	*F: 0.947 p*^*1*^*: 0.391*
RSA-Family Cohesion	18.59 ± 3.65	18.65 ± 3.73	17.06 ± 5.48	*F: 1.051 p*^*2*^*: 0.356*
RSA-Perception of Self	17.80 ± 3.87	17.70 ± 3.42	19.03 ± 3.99	*H: 3.429 p*^*3*^*: 0.180*
RSA-Social Competence	18.35 ± 3.34	17.92 ± 4.15	19.23 ± 3.86	*F: 1.045 p*^*1*^*: 0.355*
RSA-Social Resources	23.04 ± 2.84	22.16 ± 3.98	22.48 ± 3.86	*F: 0.714 p*^*2*^*: 0.494*
RSA-Total	99.96 ± 10.87	97.22 ± 14.16	99.84 ± 16.96	*H: 1.227 p*^*3*^*: 0.542*

*p*^*1*^, one-way ANOVA; *p*^*2*^, Welsch’s test; *p*^*3*^, Kruskal–Wallis’s test

### Caregiving burden, support, and informational needs in HCW relatives of cancer patients

Among the HCWs interviewed, 73.9% (*n* = 34) reported feeling an additional burden due to their profession, while 26.1% (*n* = 12) did not. Among those who reported experiencing an additional burden, the most commonly cited areas of difficulty included quick access to the treatment team (73.91%, *n* = 34), organization and execution of in-hospital tasks (71.74%, *n* = 33), questions related to the disease process (69.57%, *n* = 32), and the rapid control of pain symptoms (65.21%, *n* = 30). Additionally, 13.04% (*n* = 6) reported other burdens.

Participants were assessed regarding their experience with psychological support, information about oncological treatment, and the type of support they provided to relatives undergoing cancer treatment. Only a small proportion of HCWs (4.35%, *n* = 2) reported having previously received psychological support related to the oncological treatment process, while none of the non-HCWs (0%, *n* = 0) had received such support. Regarding information about the oncological treatment process, 19.57% (*n* = 9) of HCWs reported being informed, compared to only 8.11% (*n* = 3) of non-HCWs. Despite this, the majority expressed a desire to receive information about the treatment process. Among HCWs, 91.30% (*n* = 42) preferred to receive information, compared to 72.97% (*n* = 27) of non-HCWs. Regarding the specific aspects of support provided, the most common forms of assistance included access to medical care (91.30%, *n* = 42 of HCWs; 29.73%, *n* = 11 of non-HCWs), transportation to the hospital (84.78%, *n* = 39 of HCWs; 72.97%, *n* = 27 of non-HCWs), and financial support (41.30%, *n* = 19 of HCWs; 37.84%, *n* = 14 of non-HCWs). Experiences of burden and support in HCW and non-HCW caregivers of cancer patients are presented in Table 5.

**Table 5:** Experiences of burden and support in healthcare worker and non-healthcare worker caregivers of cancer patients

	*n* (%)
Do you feel that being a healthcare worker adds an extra burden while caring for your relative with cancer? • Yes • No	34 (73.9%) 12 (26.1%)
If you feel an additional burden, in which areas do you experience difficulties? • Quick access to the treatment team • Organizing and managing hospital-related tasks • Answering questions related to the disease process • Managing the rapid control of pain symptoms • Other difficulties	34 (73.91%) 33 (71.74%) 32 (69.57%) 30 (65.21%) 6 (13.04%)
Did the patient receive cancer treatment at the same healthcare facility where you work? • Yes • No	39 (84.78%) 7 (15.22%)
Have you, as a healthcare worker, ever received psychological support related to the cancer treatment process? • Yes • No	2 (4.35%) 44 (95.65%)
Have non-healthcare worker caregivers received psychological support related to the cancer treatment process? • Yes • No	0 (0%) 37 (100%)
Were you informed about the cancer treatment process? (Healthcare Workers) • Yes • No	9 (19.57%) 37 (80.43%)
Were you informed about the cancer treatment process? (Non-Healthcare Workers) • Yes • No	3 (8.11%) 34 (91.89%)
Would you like to receive more information about the cancer treatment process? (Yes) • Healthcare Workers • Non-Healthcare Workers	42 (91.30%) 27 (72.97%)
Did you provide support to your relative with cancer? (Yes) • Healthcare Workers • Non-Healthcare Workers	46 (100%) 33 (89.19%)
What types of support did you provide? (Healthcare Workers) • Medical care coordination • Transportation to the hospital • Financial support • Other types of support	42 (91.30%) 39 (84.78%) 19 (41.30%) 2 (4.35%)
What types of support did you provide? (Non-Healthcare Workers) • Medical care coordination • Transportation to the hospital • Financial support • Other types of support	11 (29.73%) 27 (72.97%) 14 (37.84%) 6 (16.22%)

*n*, number of persons; *%*, percent

## Discussion

Although cancer caregiving has been widely studied, this is the first to directly compare HCW and non-HCW caregivers, revealing unique challenges for HCWs [17]**.** Both groups showed higher depression and anxiety than healthy controls, with no significant differences in burnout or resilience. Many HCW caregivers reported an additional burden from their professional roles, especially in clinical management. Despite this, caregivers rarely used psychological support but expressed a strong need for treatment-related information. HCWs also provided more medical and logistical support, reflecting their professional background.

### Providing adequate information and support to cancer caregivers

This study highlights the need to recognize the informational needs of caregivers within the framework of fundamental human rights. According to Article 25 of the Universal Declaration of Human Rights, every individual has the right to a standard of living that ensures their health and well-being, including access to medical care and social support [18]. Similarly, the Convention on Human Rights and Biomedicine states that no medical intervention should occur without the individual’s free and informed consent, which requires clear communication regarding the nature, risks, and implications of the intervention [19]. The right to refuse information is as important as the right to receive it; however, without refusal, the ethical duty to inform extends to patients and their primary caregivers due to their psychological and practical interdependence. The literature increasingly highlights unmet informational needs of cancer caregivers. A study from Singapore showed most caregivers actively seek cancer-related information, especially on treatment and management, but face barriers accessing reliable sources via HCWs, often turning to the internet despite concerns about accuracy [20]. Similarly, research in Brazil found significant knowledge gaps among patients and caregivers about end-of-life issues like palliative care and advance directives. Only a minority felt adequately supported or informed by medical teams, with longer caregiving linked to higher burden [21]. These findings align with our study, which revealed a substantial unmet need for information and limited psychological support during treatment. Collectively, these studies emphasize the urgent need for comprehensive communication and systematic support to help caregivers manage the emotional and practical challenges in oncology.

### Examining the caregiving experience of HCWs

Healthcare workers, who have extensive knowledge about human health as a result of their training and have made it their profession to use this knowledge for the benefit of the patient, also can be included in this system as caregivers when their relatives receive a disease diagnosis that requires progressing with a multidisciplinary approach covering the diagnosis and treatment process.

The experiences, habits, and expectations of HCWs, who are tasked with providing healthcare in their professional lives, regarding receiving healthcare or helping their relatives who receive healthcare may vary. This variability may stem from encountering a different situation than what they apply in their own practice or from the difference between their expectations regarding the treatment process and the actual experience of this process [22].

There are positive and negative aspects of a HCW’s role as a caregiver [22, 23]. Having accurate scientific knowledge about the disease, medical ethics rooted in Hippocratic teachings, social proximity to the treatment team, and experience with disease-related situations are positive aspects. Negative aspects include the added physical, psychological, and social burdens of caregiving on top of healthcare work, potential burnout, unrealistic expectations to perform tasks usually done by the treatment team, and the challenge of balancing dual roles as caregiver and HCW [10, 24, 25]. A study conducted by Vottero and Rinke in 2021 examined the experiences of HCWs who assumed informal caregiving roles during the hospitalization of their family members. The study revealed that HCW caregivers, supported by their clinical knowledge and experience, sought active involvement in patient care and decision-making processes. However, they also experienced significant role conflict, struggling to balance their professional identity with their role as a family member, which often led to challenges in interactions with the healthcare team [23]. In line with these findings, our study demonstrated that many HCW caregivers reported feeling an additional burden due to their professional background. These results suggest that HCW caregivers face unique challenges that distinguish them from other caregivers and highlight the need for tailored psychosocial support interventions to address their specific needs.

### Expectations placed on HCWs: access to treatment and in-hospital responsibilities

In our study, among HCW caregivers who reported feeling an additional burden due to their professional identity, the most frequently cited difficulties were quick access to the treatment team and organizing in-hospital tasks. This finding may be better understood within the context of structural challenges in the Turkish healthcare system. According to 2021 data from the Organisation for Economic Co-operation and Development (OECD), while the average number of physicians per 1000 people in OECD countries is 3.7, this figure is 2.2 in Turkey, placing Turkey at the lowest rank among member countries [26]. A comparative study of 18 OECD countries (2002–2018) showed that physician consultations per capita in Turkey rose from 3.1 to 9, the highest increase among the countries. However, despite a slight rise in physician numbers, this growth did not meet demand, leading to shorter consultation times—dropping from about 50 min in 2002 to 23 min in 2018 [27].

These figures suggest that patients in Turkey, especially those with complex conditions like cancer, face barriers to timely care. Families often rely on HCW relatives to navigate the system, adding pressure on these caregivers to balance informal care with professional duties.

Beyond physician shortages and patient load, systemic issues such as poor coordination, limited administrative support, and unclear role definitions increase this burden. HCW caregivers struggle to reconcile their clinical roles with personal caregiving, leading to communication challenges with care teams [23]. Gouveia et al. also noted the emotional strain from dual expectations at work and home [28]. These findings emphasize the need for targeted interventions that address both systemic gaps and provide psychosocial support for HCW caregivers, especially in demanding settings like oncology.

### Anxiety, depression, and psychological resilience

In our study, both HCW (HCW) and non-HCW (non-HCW) caregivers exhibited significantly higher levels of anxiety and depression compared to healthy controls, while no significant difference was found between the caregiver groups themselves. However, these results should be interpreted with caution due to limitations such as the inclusion of caregivers of patients predominantly undergoing primary or adjuvant treatment and the concentration of stage IV cases in the sample.

Working life is a fundamental factor affecting individuals as biopsychosocial beings. Among working environments, hospitals—where life and death frequently intersect—present unique stressors. Previous research has shown that hospital work is particularly demanding due to constant human interaction, the need for rapid decision-making, and the high stakes of life-or-death situations, all of which HCWs routinely face [29]. The absence of a significant difference in anxiety levels between HCW (HCW) and non-HCW caregivers may be attributable to several factors. HCWs’ familiarity with high-stress situations, their higher health literacy, and the coping strategies they have developed through both professional experience and personal crises likely contribute to their ability to manage anxiety. Moreover, during interviews, HCW caregivers frequently exhibited a tendency to project a competent self-image, which may represent an internalized strategy for managing emotional challenges and maintaining psychological resilience.

Experiences shape individual responses to stress. HCWs, who are frequently exposed to death and dying, may develop more functional coping mechanisms, potentially resulting in increased resilience when caring for relatives with terminal illnesses. Personal traits common among HCWs—such as perfectionism, heightened attention to detail, self-sacrifice, and a tendency to prioritize the needs of others—are integral to professional identity and may influence caregiving experiences [30]. Furthermore, the perception of personal accomplishment is often a key component of self-worth for HCWs, and failure to achieve positive outcomes in complex cases like cancer can contribute to burnout [31].

Our study found HCW caregivers scored lower in personal accomplishment than others, reflecting higher responsibility and self-expectation. They often compensate for system gaps, increasing psychological burden and risk of burnout, low self-esteem, and depression. Due to their nature, HCWs may avoid seeking help or recognizing their distress [32, 33]. Additionally, concerns about stigma may prevent them from expressing mental health struggles or even recognizing these issues themselves. These findings underscore the need for targeted interventions to increase mental health awareness among HCWs, despite their professional knowledge and involvement in healthcare delivery [34].

In our study, no significant differences were found between HCW caregivers, non-HCW caregivers, and healthy controls regarding overall psychological resilience and its subdimensions. This finding suggests that, despite the high emotional burden and stress associated with caring for advanced-stage cancer patients, caregivers may draw on existing coping resources to maintain their resilience. For HCWs in particular, frequent exposure to crisis situations and high-stakes clinical environments may contribute to the development of adaptive coping skills and emotional regulation strategies, supporting their resilience even in personal caregiving contexts[35]. Furthermore, HCWs often face societal and professional expectations to remain strong and competent, which may lead them to suppress emotional vulnerability and influence how resilience is expressed or reported. These findings highlight the complex relationship between professional identity, caregiving responsibilities, and psychological resilience. They suggest that supporting resilience in HCW caregivers requires not only reinforcing existing coping strategies but also addressing long-term occupational stress and emotional fatigue through targeted mental health interventions [36].

## Conclusion and limitations

One of the main limitations of this study is the relatively small sample size, particularly when conducting statistical comparisons between subgroups. This limitation may affect the generalizability of the findings and the statistical power of the analyses. Therefore, all comparisons and conclusions drawn from these data should be interpreted with caution. Additionally, a significant portion of the healthy control group consisted of hospital staff, who, due to their employment in demanding clinical environments, may already experience elevated levels of occupational stress. We believe this factor could have influenced our findings, potentially contributing to the absence of significant differences in Maslach Burnout Inventory scores between the caregiver groups and the control group.

A significant difference was found between caregiver groups and healthy controls in anxiety and depression levels, aligning with existing literature and supporting our study’s purpose. However, discrepancies appeared when comparing self-report scales to clinician-administered interviews, which are the gold standard in mental health assessments. This underscores the need to use both objective clinical evaluations and subjective self-reports in future research. Qualitative studies may provide deeper insights into caregivers’ complex experiences and the psychological impact of oncology caregiving. Our findings also highlight the importance of developing strategies to identify clinical risks among caregivers and implement timely interventions.

As a conclusion, this study suggests that HCWs who also serve as caregivers may experience role conflict between their professional identity and family responsibilities, which could contribute to increased psychological burden. It was also observed that this group might have a greater need for information about the treatment process and more open communication with healthcare professionals. These findings highlight the potential importance of developing more comprehensive support systems for caregivers within oncology practice and suggest that clinicians should consider addressing the psychosocial needs of caregivers as part of holistic patient care.

## Data Availability

No datasets were generated or analysed during the current study.
